# Highly crystalline nickel hexacyanoferrate as a long-life cathode material for sodium-ion batteries

**DOI:** 10.1039/d0ra03490h

**Published:** 2020-07-21

**Authors:** Ratul Rehman, Jian Peng, Haocong Yi, Yi Shen, Jinwen Yin, Chang Li, Chun Fang, Qing Li, Jiantao Han

**Affiliations:** School of Materials Science and Engineering, State Key Laboratory for Materials Processing and Die & Mould Technology, Huazhong University of Science and Technology Wuhan 430074 People's Republic of China pj9301@hust.edu.cn jthan@hust.edu.cn

## Abstract

Prussian blue analogs (PBAs) are attractive cathode candidates for high energy density, including long life-cycle rechargeable batteries, due to their non-toxicity, facile synthesis techniques and low cost. Nevertheless, traditionally synthesized PBAs tend to have a flawed crystal structure with a large amount of [Fe(CN)_6_]^4−^ openings and the presence of crystal water in the framework; therefore the specific capacity achieved has continuously been low with poor cycling stability. Herein, we demonstrate low-defect and sodium-enriched nickel hexacyanoferrate nanocrystals synthesized by a facile low-speed co-precipitation technique assisted by a chelating agent to overcome these problems. As a consequence, the prepared high-quality nickel hexacyanoferrate (HQ-NiHCF) exhibited a high specific capacity of 80 mA h g^−1^ at 15 mA g^−1^ (with a theoretical capacity of ∼85 mA h g^−1^), maintaining a notable cycling stability (78 mA h g^−1^ at 170 mA g^−1^ current density) without noticeable fading in capacity retention after 1200 cycles. This low-speed synthesis strategy for PBA-based electrode materials could be also extended to other energy storage materials to fabricate high-performance rechargeable batteries.

## Abstract 2

A low-speed synthesis strategy was designed to fabricate Prussian blue analog based electrode materials for high-performance rechargeable batteries.

## Graphical abstract

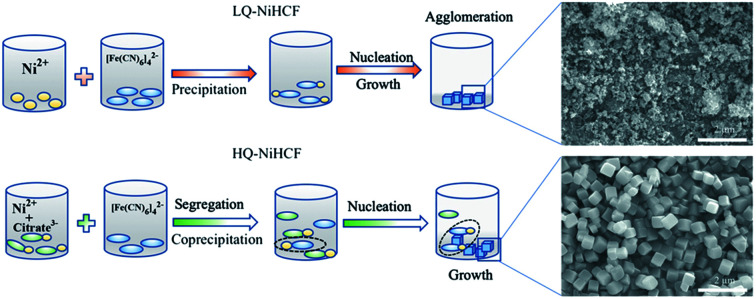

## Introduction

Lithium-ion batteries (LIBs) are well known in application and are gaining significant interest for energy-storage applications.^1–5^ Although LIBs have numerous advantages, such as long cycling life, high recycling efficiency and high energy density, which meet the necessary requirements for electrical energy storage systems (EESs), the cost of lithium is one point that ought to be given more consideration from the perspective of large-scale usage. Latterly, the cost of lithium has been escalating rapidly, owing to the irregular circulation of worldwide lithium reserves.^6^ To address this challenge, sodium-ion batteries (SIBs) have become a progressively more appealing option to LIBs in EESs because of their low cost and elongated lifecycle.^7,8^ Besides, the chemical attributes of sodium show similar properties and comparing its source, sodium has more plentiful sources than lithium, and thus different types of cathode materials have been contemplated for SIBs.

In recent years, metal–organic frameworks (MOFs) have been prevalent in considering this purpose.^9–11^ Because of their moderately low capacity value,^12,13^ including poor rate performance,^14^ and competition with their anode counterparts,^15–17^ cathode materials are of immense significance in hastening the commercialization of SIBs. However, a significant distortion of and strain on the crystal lattice may be induced due to the broad radius of Na^+^ during the incorporation phase, and this also poses challenges in finding relevant electrode materials for SIBs.^18–21^ Some cathodes, P_2_–Na_0.6_7Co_1−*x*_Ti_*x*_O_2_,^22^ Na_3_V_2_(PO_4_)_3_,^23,24^ NaTi_2_(PO_4_)_3_,^25,26^ and Na_0.7_[Mn_1−*x*_Li_*x*_]O_2+*y*_,^27^ demonstrated significant performance in terms of variable capacity, including rate efficiency, but due to certain phase transitions, and large changes in volume during cycling, they have failed to meet long cycling-life requirements. Prussian blue analogs (PBAs), a kind of metal–organic framework with the formula A_*x*_M_*y*_[M′(CN)_6_]_*z*_·*n*H_2_O, where A refers to an alkaline metal and M, M′ refer to transition metals, have been widely studied as attractive cathodes for SIBs in the past decade due to their high theoretical capacity and long-cycle life. As a significant cathode source for Na-ion batteries, PBAs have been given a lot of attention, not only for their rigid open framework (which can ensure suitable Na^+^ flexibility with large-scale interstitial sites) but also since they present a volume difference between Na^+^ insertion and extraction.^28–31^ Also, their facile synthetic procedure makes them suitable for large-scale applications, besides their nontoxicity and low cost, and they have become more widespread.^32,33^ The open crystal structure could effectively tolerate flexibility in the lattice volume and facilitate the passage of Na^+^.^34–40^

However, the theoretical value of specific capacity for PBAs is still unreachable, apart from their poor cycling stability and low coulombic efficiency, due to defects and crystal water in the framework of PBAs.^34,41,42^ Earlier it was proved that the electrochemical performance of PBAs is firmly identified with their inherent crystal structure.^43,44^ In our research group, synthesized CeHCF as a cathode material performed with a specific capacity of ∼60 mA h g^−1^ at 0.25C (with capacity retention of about ∼80% at 8.3C) in LIBs, and similarly ∼55 mA h g^−1^ at 0.25C (with capacity retention of nearly 55% at 8.3C) in SIBs, due to an open network with large lattice spaces.^45^ Additionally, recently some innovative work has reported on PBAs based upon long-term cyclability and improved electrochemical kinetics as well efficiency.^46–49^ Guo's group revealed that the suitability of K_0.09_Ni[Fe(CN)_6_]_0.71_·6H_2_O as a cathode for SIBs was attributed to its zero-strain characteristics, where there was less than 1% change in lattice parameter distortion during the Na^+^ insertion/extraction phase^50^ and it achieved excellent cycling stability and a highly stable structure. Jiang's group also reported remarkable cycling stability from synthesized Na_1.014_Ni[Fe(CN)_6_]_0.818_·3.53H_2_O *via* a simple co-precipitation method, but the specific capacity was as low as 68 mA h g^−1^.^51^ Nevertheless, in practical applications, the tested A_*x*_Ni[Fe(CN)_6_]_*y*_ cathodes could not carry more sodium content and presented a high number of defects in crystallization. To increase the content of sodium in the framework, Liu's group firstly came up with the idea where different amounts of sodium citrate were employed in the synthesis process as a cathode material for SIBs.^52^ Recently, another study has been done on highly stable monoclinic sodium-rich nickel hexacyanoferrate by introducing tri-sodium citrate to control its crystallization.^53^ Henceforth, when designing a new cathode, it is important that the sodium content should be high with fine crystallization and meet the requirements of applications, such as temperature adaptability, steadiness and more. The preparation of nickel–iron Prussian blue analogs (Ni–Fe PBAs) has often shown a significant amount of vacancies within the crystal structure due to quick precipitation *via* the conventional synthesis process.^54^

In this research, we have established low-defect and sodium-rich nickel hexacyanoferrate nanocrystals synthesized through a simple co-precipitation mechanism with the assistance of a chelating agent called tri-sodium citrate. Because of the moderate development process for crystallization, HQ-NiHCF displayed a small zeolite water content with few [Fe(CN)_6_]^4−^ openings in the crystal structure. For these reasons, the ion storage ability was significantly improved and there were adequate transportation behaviors for Na^+^ and e^−^, as well as effective preservation of the integrity of the crystal structure during cycling. Hence, in terms of its good specific capacity of nearly 80 mA h g^−1^, which is very close to the theoretical value, remarkable cycling stability of 78 mA h g^−1^ around 1200 cycles, and superb rate capability of 62 mA h g^−1^ at 10C with high coulombic efficiency, it has shown persuasive electrochemical performance as a cathode for SIBs. For comparison purposes, low-quality nickel hexacyanoferrate (LQ-NiHCF) was also synthesized by the conventional procedure without any additive, and the relative performances are offered in our report.

## Experimental

### Synthesis route of HQ-NiHCF and LQ-NiHCF

The HQ-NiHCF PBA was synthesized by an enhanced co-precipitation technique where we added tri-sodium citrate (Na_3_C_6_H_5_O_7_·2H_2_O) as a chelating agent.^52^ To make solution A, 5 mmol of NiCl_2_·6H_2_O and 5 mmol of Na_3_C_6_H_5_O_7_·2H_2_O were dissolved in 50 ml of distilled water. Afterward, to prepare solution B, 5 mmol of Na_4_Fe(CN)_6_ was dissolved in 50 ml of deionized water. Finally, to form solution C, 0.5 g of polyvinylpyrrolidone K-30 (PVP) was gradually mixed in 200 ml of deionized water. Then solutions A and B were added directly to solution C under nonstop stirring for 2 h, and aged at room temperature for 24 h. From that point onward, the sediment was gathered by centrifugation and washed with deionized water several times (minimum 3 times) and washed with ethanol once. The sediment was eventually dried in a vacuum oven at 100 °C for 24 h and the HQ-NiHCF PBA was collected.

In general, the synthesis of LQ-NiHCF PBA was done by the precipitation method. Typically, 5 mmol of NiCl_2_.6H_2_O was dissolved in 50 ml of deionized water to develop solution A. Then a 5 mmol volume of Na_4_Fe(CN)_6_ was mixed in 50 ml of deionized water to get solution B. Finally, to produce solution C, 0.5 g of PVP K-30 was mixed in 200 ml of deionized water until it dissolved. Now, by using a peristaltic pump, solution (A) and (B) were added to solution (C) until there was no dribbling, at which point it was kept under stirring for 6 h, then aged for 24 h and similar procedures were pursued to those above to obtain samples.

### Materials characterization

The XRD patterns were recorded with a Panalytical X'pert PRO MRD (Holland) using Cu Kα radiation. Scanning electron microscopy (SEM, TESCAN VEGA3) and transmission electron microscopy (TEM, JEM-2100 electron microscope) were used to analyze the morphologies of the samples as well as their sizes. The thermal evaluation curve was analyzed with a thermogravimetric analyzer (TGA, Netzsch STA 449 F3) under an air atmosphere from room temperature to 620 °C at a heating rate of 10 °C min^−1^. The elements were identified using an elemental analyzer (Vario Micro), and the chemical composition of the Fe, Ni and Na elements was studied by inductively coupled plasma optical emission spectroscopy (ICP-OES, IRIS Intrepid II XSP, Thermo Elemental, USA).

### Electrochemical measurements

The working electrodes were designed by mixing the active ingredients, Ketjen black and polytetrafluoroethylene in a weight ratio of 7 : 2 : 1 to form a slurry, transferred to an aluminium mesh, and eventually pressed to fabricate a thin film, then vacuum dried at 100 °C. To assess electrochemical efficiency, a regular coin-cell (CR2032) was assembled where metallic sodium was used as the counter-electrode. For an electrolyte, 1.0 mol L^−1^ NaClO_4_ in a solution of ethylene carbonate/diethyl carbonate (1 : 1 vol) with 2 wt% fluoroethylene carbonate was used with Whatman glass fibers as a separator. Each of the cells was installed in an Ar-filled glove box at room temperature. The cyclic voltammetry (CV) curves were measured on an electrochemical workstation (Princeton) at 0.1 mV s^−1^ and the galvanostatic charge/discharge experiments were carried out on a battery testing system (LAND cycler, Wuhan Kingnuo Electronic) from 2.0 to 4.2 V *versus* Na^+^/Na.

## Results and discussion

### Structure analysis and chemical properties

**Fig. 1:**
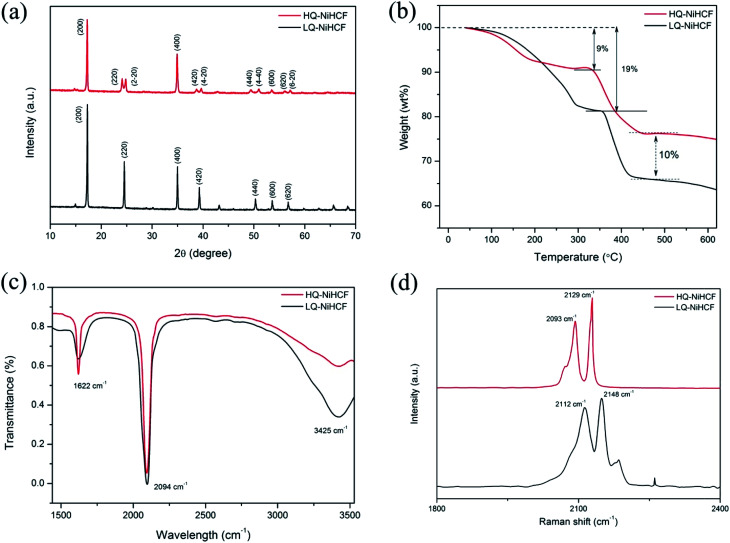
(a) XRD patterns of the as-prepared HQ-NiHCF and LQ-NiHCF; (b) thermogravimetric curves; (c) Fourier-transform infrared spectroscopy (FT-IR) pattern; and (d) Raman spectra of HQ-NiHCF and LQ-NiHCF samples.

According to the XRD results in Fig. 1a, we noted that the typical PBAs achieved a face-centered cubic structure.^55^ The peaks (220), (420), (440) of HQ-NiHCF were split into almost equal forces which was due to the expansion of the crystal lattice and decrease in symmetry caused by the large amount of sodium ions in the crystal structure. The enhanced crystallinity was mainly due to the low-speed co-preparation methods, resulting in an intact crystal.^11,34,56,57^ Through the conventional precipitation process, LQ-NiHCF suffered very poor crystallization. The reason is that the growth stage and nucleation of Na_2_Ni[Fe(CN)_6_] happened immediately, later causing a large amount of Fe(CN)_6_ defects, and synchronized water molecules in the crystal framework. However, in the chelating agent-assisted co-precipitation method, tri-sodium citrate (Na_3_C_6_H_5_O_7_·2H_2_O) was co-precipitated with a solution of sodium ferrocyanide, which basically suppressed the rate of growth and achieved a superbly crystallized Na_2_Ni[Fe(CN)_6_] sample. The formulae of LQ-NiHCF PBAs and HQ-NiHCF PBAs were determined to be Na_0.79_Ni[Fe(CN)_6_]_0.74_, and Na_1.67_Ni[Fe(CN)_6_]_0.87_, corresponding to 26% and 13% defect contents, respectively.

The amount of water content and the material degradation process were studied by TGA, as shown in Fig. 1b. Here, there were noticeable weight losses of 9% and 19% below a temperature of 200 °C for HQ-NiHCF and LQ-NiHCF, respectively, resulting from the weight loss of water content in the sample, attributed to adsorbed water loosely bonded with the particle surfaces, including the frameworks.^58,59^ There was less weight loss for HQ-NiHCF because of its synthesis method where water coordination was reduced. Furthermore, the final material degradation for both materials at high temperature (>500 °C) showed that the newly fabricated samples were almost 10% more efficient than the generally synthesized LQ-NiHCF. The chemical bonds were examined in the structures and the presence of contamination was determined using Fourier-transform infrared (FT-IR) spectra.^60^ From the position of the absorption peaks for these two samples in Fig. 1c, it can be seen that both samples were consistent with indications of the existence of the same functional groups. The strong absorption peak at 2094 cm^−1^ corresponded to the elongating vibrations of the C≡N ligands coordinated with Fe^2+^ and Ni^2+^.^61^ The absorption points at 1622 cm^−1^ and the area between 3300 and 3500 cm^−1^ could be ascribed to the O–H bending vibrations and stretching O–H modes, respectively, due to interstitial water.^62^

Raman spectroscopy was used to explain the change in valance state of iron in the compound, as shown in Fig. 1d. Both samples achieved almost the same peaks in the diagram. The two peaks traced at 2093 and 2129 cm^−1^ for HQ-NiHCF and the peaks at 2112 and 2148 cm^−1^ for LQ-NiHCF, are attributed to the vibrations of the cyanide group and show that Fe and Ni ions exhibited divalent ions and almost two Na ions existed in the materials (Fe^II^–C≡N–Ni^II^).^63^ According to the increased intensity of the peaks, it can be speculated that the number of Na ions increased with the slow reaction rate in the synthesis of HQ-NiHCF.

**Fig. 2:**
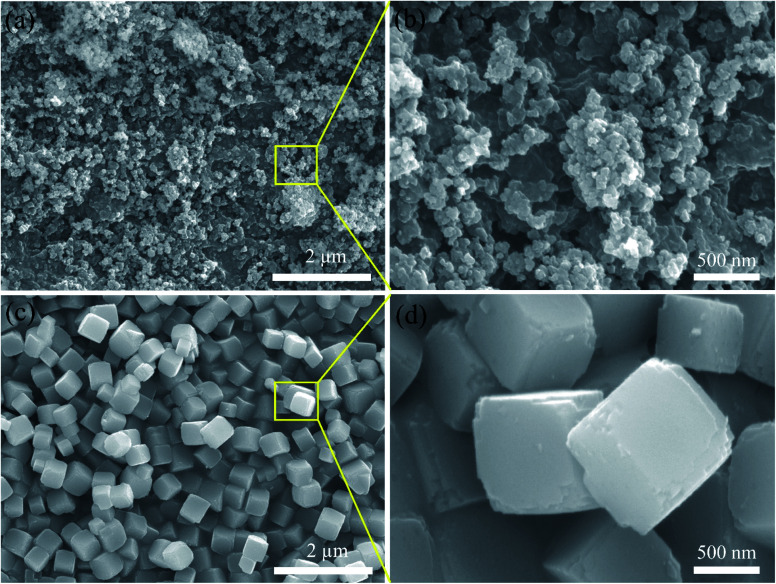
SEM images at different magnifications of (a) and (b) LQ-NiHCF; (c) and (d) HQ-NiHCF.

**Fig. 3:**
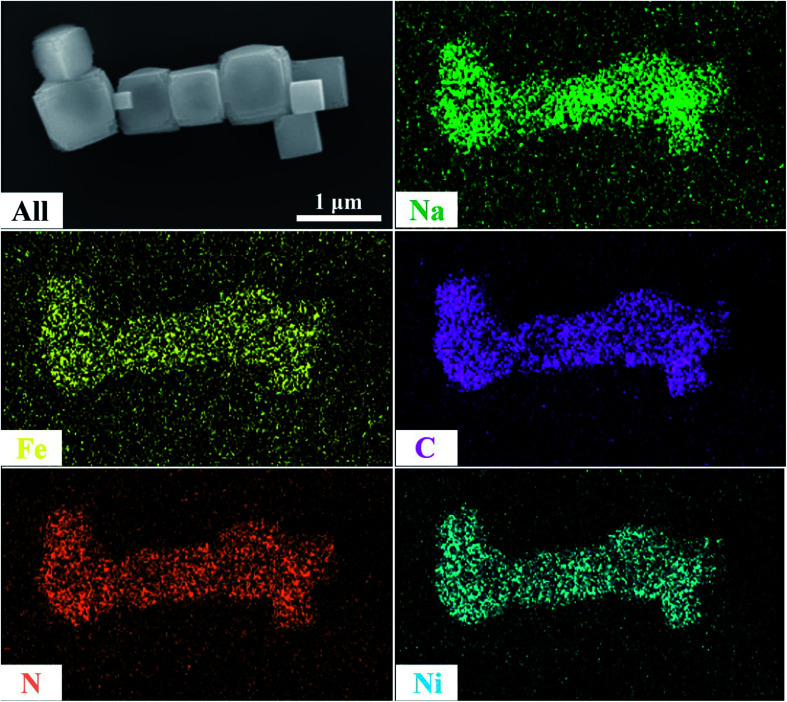
Energy dispersive spectroscopy (EDS) mapping images of HQ-NiHCF.

**Fig. 4:**
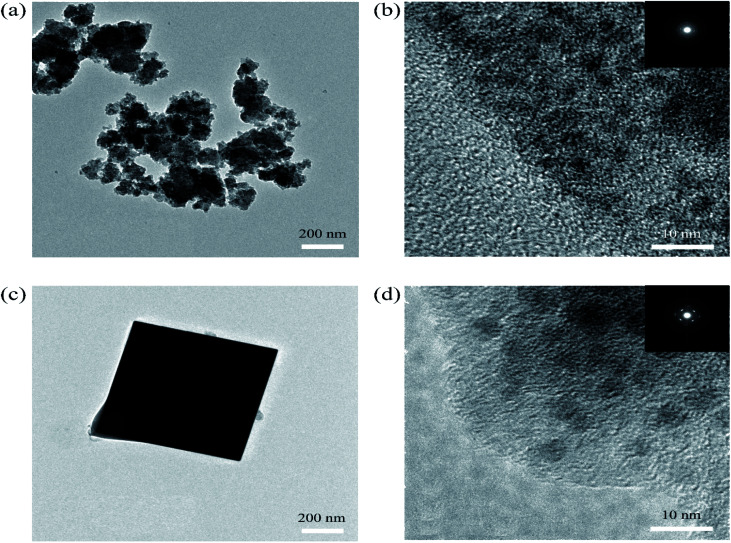
(a) and (b) TEM images of the polycrystalline LQ-NiHCF PBA with SAED patterns (inset); (c) and (d) TEM images of single-particle HQ-NiHCF PBA nanocrystals with SAED patterns (inset).

SEM was used to explain the size of the nanoparticles of the as-prepared samples. Here, the different magnification images of the LQ-NiHCF sample shown in Fig. 2a and b were composed of erratically rough agglomeration particles due to the conventional precipitation processes where the crystallization and growth occurred so fast.^44^ In contrast, due to the deceleration and segregation of crystal growth, HQ-NiHCF in Fig. 2c and d displays uniform nanocubes. With the assistance of the chelating agent, the nucleation kinetics and crystal aggregation are reduced. By using energy dispersive spectroscopy (EDS) mapping, Na, Fe, and Ni elements were confirmed in the HQ-NiHCF samples, as shown in Fig. 3. Moreover, its high purity and integrity can be suggested, as these components are homogeneously distributed throughout the materials.^34^ The TEM images are shown in Fig. 4, where in Fig. 4(a and b) it can be seen that the LQ-NiHCF sample was polycrystalline. The sample HQ-NiHCF presented an electron diffraction pattern showing single particle nanocrystals, and the SAED patterns (as inset in Fig. 4b and d) also supported the high crystallinity of the samples, as shown in Fig. 4(c and d). It can be perceived that this kind of nano-cubic structure could be very suitable for Na^+^ and e^−^ transportation in the electrochemical reaction phase.

**Scheme 1:**
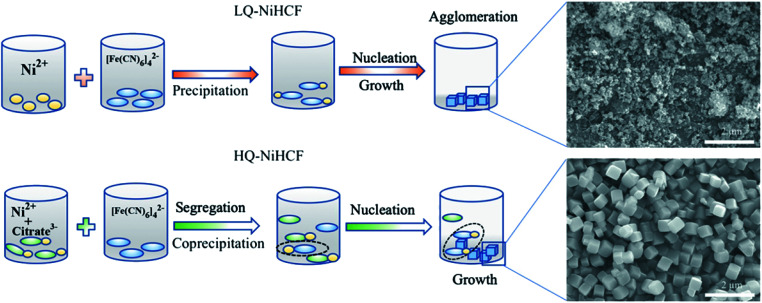
Schematic illustration of the conventional method of precipitation for LQ-NiHCF and the chelating agent-assisted co-precipitation method which controls the process of crystallization and synthesis for HQ-NiHCF.

Based on the aforementioned experimental data, a probable mechanism for the growth of HQ-NiHCF nanocubes is suggested and shown in Scheme 1. In general, in the conventional precipitation process synthesis, Ni^2+^ cations responded to [Fe(CN)_6_]^4−^ anions in the aqueous solution. Here, the nucleation and growth stage happened instantly, and therefore the particles accumulated together, with large amounts of [Fe(CN)_6_]^4−^ flaws also corresponding to water inside the framework. Citrate, by playing the main role in controlling the speed of agitation and segregating molecules, helped form perfectly shaped nanocubes with prominently inhibited [Fe(CN)_6_]^4−^ defects. In the chelating agent-assisted method, the Ni^2+^ ions firstly linked with citrate ions and formed a nickel-citrate chelate, and afterward co-precipitated in aqueous solution with hexacyanoferrate ions. The nickel-citrate chelate served as a buffer for gradually releasing Ni^2+^ ions to interact with hexacyanoferrate ions to form initial nucleuses. Due to the high multifaceted potential of citrate ions and their carrying a negative charge, they accumulated uniformly on the nucleus surfaces, thereby overturning the rate of growth and suppressing the aggregation of crystals. Eventually, the slow growth and constant release of Ni ions helped these nucleuses to develop well-formed monodispersed Prussian blue nanocubes with high crystallinity.

### Electrochemical properties testing

**Fig. 5:**
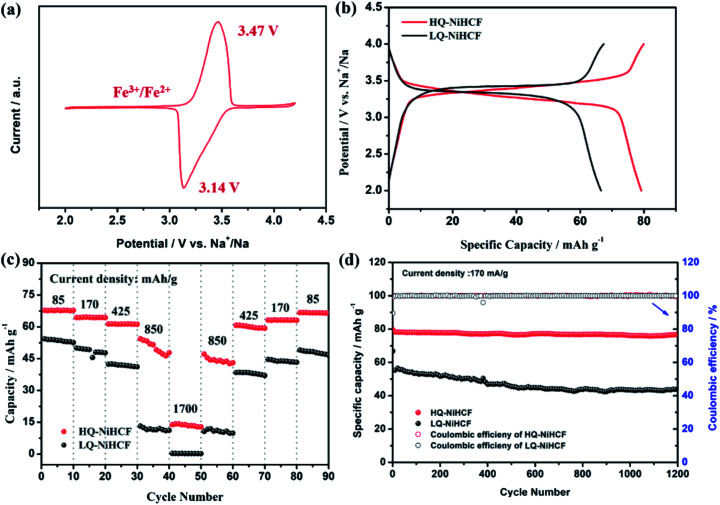
Electrochemical properties of HQ-NiHCF and LQ-NiHCF: (a) Typical CV curves measured at a scan rate of 0.1 mV s^−1^; (b) charge/discharge voltage profiles at a current density of 15 mA g^−1^; (c) rate performance at different current density ranges; (d) cycle performance at a current density of 170 mA g^−1^.

**Fig. 6:**
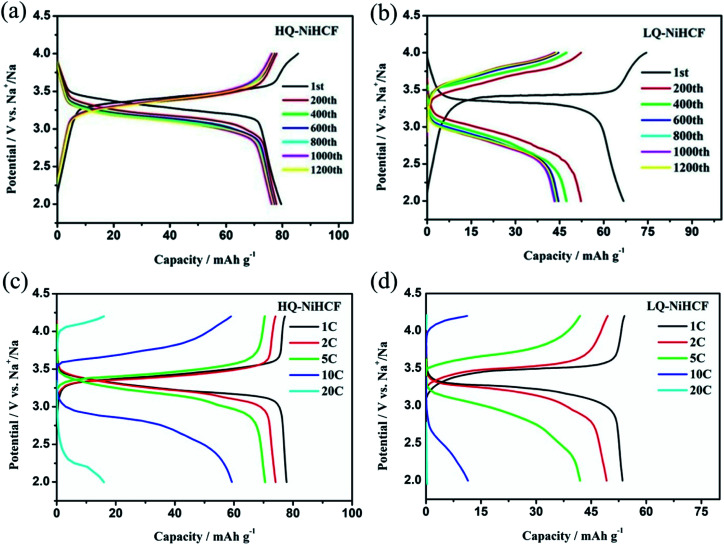
Cycle performance of: (a) HQ-NiHCF; (b) LQ-NiHCF, and the rate performance of (c) HQ-NiHCF; (d) LQ-NiHCF.

Electrochemical performances were studied by galvanostatic charge/discharge and cyclic voltammetry (CV). Fig. 5a shows the CV of HQ-NiHCF at a scan rate of 0.1 mV s^−1^ within 2.0 and 4.2 V (*vs.* Na^+^/Na) where a pair of distinct reduction/oxidation peaks appeared at 3.47/3.14 V, correlating with the balance potential of Fe^2+^/Fe^3+^;^64^ Ni^2+^ remains electrochemically inert for the sodium reaction.^31,65^ From Fig. 5b it can be seen that the initial charge and discharge capacities of HQ-NiHCF are 79.93 and 79.19 mA h g^−1^, respectively, at a current density of 15 mA g^−1^, which is consistent with a coulombic efficiency of almost 99% while LQ-NiHCF delivers lower charge/discharge capacities of 67.43 and 66.55 mA h g^−1^, respectively. Here, Na^+^ storage sites were utilized more in HQ-NiHCF, and thus it can be ascribed to the high crystallinity and reduced water content inside the crystal framework together with a lower number of [Fe(CN)_6_] vacancies.^34,66^ In the fast charge/discharge process, HQ-NiHCF achieves an enhanced rate capability. Fig. 5c shows the rate performance of HQ-NiHCF and LQ-NiHCF, where HQ-NiHCF shows a higher overall specific capacity than LQ-NiHCF, and the specific discharge capacities of HQ-NiHCF are 78, 74, 70, 63 mA h g^−1^ from 85 to 850 mA g^−1^, which remained incredibly stable without any obvious capacity loss over 90 cycles once the current changed back to 85 mA g^−1^. However, LQ-NiHCF could not hold any capacity and only maintained ∼47 mA h g^−1^ at 425 mA g^−1^. In practical applications, the cycling performance and the coulombic efficiency play an endlessly significant role.^67^ Moreover, HQ-NiHCF showed a dramatically improved cycling performance compared to LQ-NiHCF. Fig. 5d presents the long cycling behavior of HQ-NiHCF beneath a current density of 170 mA h g^−1^ and LQ-NiHCF was also tested for comparison. Although LQ-NiHCF performed with excellent cyclic stability but a low discharge capacity of 57 mA h g^−1^, it exhibited a declining drift with a very poor coulombic efficiency. Remarkably, the HQ-NiHCF cathode carried a high discharge capacity of 78 mA h g^−1^ and capacity retention of ∼98% after 1200 cycles. While charging, a side reaction happened with zeolite water, and therefore the coulombic efficiency was a little lower for only the first few cycles, but later on, it jumped to more than 98%, which is considerably more than that of LQ-NiHCF, as shown in Fig. 6a and b. The capacity retention at different current densities was also observed, through long-term cycling, as shown in Fig. 6c and d, where it is clearly indicated that HQ-NiHCF held its capacity of almost 62 mA h g^−1^ at 10C but LQ-NiHCF retained only 13.7 mA h g^−1^.

Such an improvement in the efficiency of Na-storage could be associated with Na-ion intercalation habits, the lower amount of water and the resulting improved crystallinity of HQ-NiHCF. These findings specify that the physical steadiness of the PBAs can be considerably improved by enriching the quality of the crystal structure. The abovementioned finding also verifies that the advent of PBAs with high-quality crystal structures will lead to progress in their applications.

## Conclusions

In summary, we have effectively fabricated low-defect and Na-rich nickel hexacyanoferrate nano-particles by introducing a chelating agent. Here, tri-sodium citrate acted to slow down the crystallization speed and achieved perfectly shaped crystalline particles. Due to the suppressed amount of [Fe(CN)_6_]^4−^ vacancies, including water molecules, in the framework, the HQ-NiHCF PBA exhibited a high specific capacity of 80 mA h g^−1^ at 15 mA g^−1^, and the cycling stability after 1200 cycles still remained at 99%, and 78 mA h g^−1^ of the capacity delivered at a current density of 170 mA g^−1^ and an excellent rate capability of 63 mA h g^−1^ at 850 mA g^−1^. Although it showed a lower specific capacity, considering its excellent electrochemical performance, simple method of synthesis and low cost, the application of HQ-NiHCF is a promising cathode material for SIBs in the large-scale energy storage systems.

## Conflicts of interest

There are no conflicts to declare.

## Supplementary Material
